# Regional Disparities in Growth Patterns of Children with Cerebral Palsy: A Comparative Analysis of Saudi Arabian, UK, and US Data

**DOI:** 10.3390/children11080891

**Published:** 2024-07-25

**Authors:** Mshari Alghadier, Reem M. Basuodan, Reem A. Albesher, Saadia Waqas, Eman Misbah Suliman, Mohammed Hassan

**Affiliations:** 1 Department of Health and Rehabilitation Sciences, Prince Sattam bin Abdulaziz University, Alkharj 11942, Saudi Arabia; 2 Department of Rehabilitation Science, Princess Nourah bint Abdulrahman University, Riyadh 11671, Saudi Arabia; rmbasoudan@pnu.edu.sa (R.M.B.); raalbesher@pnu.edu.sa (R.A.A.); 3 School of Health Sciences and Social Work, Griffith University, Nathan, QLD 4111, Australia; saadia.waqas@griffithuni.edu.au; 4 Children with Disability Association, Riyadh 12273, Saudi Arabia; e.misbah@dca.org.sa (E.M.S.); dr-mohammed@dca.org.sa (M.H.)

**Keywords:** cerebral palsy, neurodevelopmental disorder, gross motor function classification system (GMFCS), Saudi Arabia, growth patterns

## Abstract

Aim: In order to understand the global variations in the growth trajectories of cerebral palsy patients, this study aimed to compare the growth patterns of cerebral palsy patients in Saudi Arabi with United States and United Kingdom counterparts. Method: Anthropometric data from 107 participants with cerebral palsy in Saudi Arabia were collected, including age, gender, cerebral palsy type, Gross Motor Function Classification System level, birth weight, weight at assessment, height at assessment, body mass index, and head circumference at assessment. Results: This study found discrepancies between the growth patterns of Saudi Arabian children with cerebral palsy and United Kingdom and the United States growth charts, particularly among those with severe cerebral palsy. Significant differences were observed in weight, height, and body mass index z-scores when comparing Saudi Arabian data with the United kingdom and United States reference data. Interpretation: These findings emphasize the importance of validating growth charts across different populations to ensure accurate monitoring and clinical management of children with cerebral palsy. Additionally, this study highlights the need for region-specific growth references to better address the diverse needs of individuals with cerebral palsy worldwide.

## 1. Introduction

Cerebral palsy (CP) is a severe childhood neurological disorder with an incidence of approximately 1.6 per 1000 live births in high-income countries [1]. In Arabic-speaking regions, the incidence is slightly higher, at approximately 1.8 per 1000 live births [2]. Although CP is primarily characterized by postural and motor disturbances, it also encompasses a variety of problems, including perceptual, cognitive, communicative, emotional, and behavioral difficulties [3,4,5]. These complications limit the activities and participation of affected children. Consequently, children with CP and their families experience significant stress [4,6,7]. Compared to those without impairments or chronic illnesses, they can have reduced access to healthcare [8], and have to wait a long period to receive the treatments they require [9,10].

There has to be more clarity in the current recommendations for giving children with CP high-quality treatment [11,12]. Many of the present suggestions might not be sufficient to fulfill the requirements of children and their families due to a lack of input from parents and children [11]. However, in order to provide the greatest care for children with CP, we must first comprehend the traits and resources available to these children as well as the families. Services available to children with CP may vary by child and family [13]. Furthermore, the availability and quality of physical therapy services for children living in different geographic areas within the same country may vary [12].

Research has shown that the motor function of children with CP is important in determining the types of services they receive and also affects the needs and concerns of parents [14]. Additionally, a variety of demographic characteristics and limited access to rehabilitation services were found to be significantly correlated by Al-Imam et al. (2021) [13]. The Gross Motor Function Classification System (GMFCS) III–V levels, hearing loss, significant mobility challenges, parents with less education, female gender, and lower family income were some of these characteristics. According to Ryan et al. (2021) [15], there are a number of characteristics that affect how often young outpatients with CP in England—ages 10 to 19—use health and rehabilitation facilities. Their results showed that the most common specialists were physiotherapists, followed by dentists, general practitioners, occupational therapists, and podiatrists. Factors such as age, GMFCS, and language impairment have been found to influence service use.

In a study conducted in the United Kingdom (UK) and the United States (US), Wright et al. (2017) [16] summarized the growth trajectory of children with CP using large datasets from the two countries. The study analyzed data from more than 24,000 California patients between the ages of 2 and 20, classified into five severity levels based on the GMFCS. These charts are an important tool for monitoring the growth of children with CP and assessing deviations from typical growth patterns. More specifically, the aim of the study was to assess how well growth data for children with CP in the UK fit these curves compared to traditional regional growth curves used in the UK. A new analysis of US CP trends using Lambda Musigma (LMS) allowed researchers to calculate the standard deviation of z-scores and assess ability data from 195 children with CP in Glasgow, UK. The results showed significant differences in the growth patterns of patients with CP in the UK, WHO data, and patients in the US, especially in children with severe CP, highlighting the need to test growth patterns in different populations to ensure appropriate clinical follow-up and treatment.

There is a dearth of epidemiological data on CP in Arabic-speaking nations [2,17,18]. Saleh and Almasri [17] provided details of a service in Jordan for children with CP between the ages of one and seventeen. According to their findings, the most frequent type of CP, accounting for 74.1% of cases, was spastic CP. The most frequent sequelae were speech and visual problems. Moreover, with a 90.4% utilization rate, physical therapy is the most popular service for children with CP in Jordan. Family resources, children’s special needs, related difficulties, and general satisfaction with services are among the factors that impact the utilization of services in Jordan [19].

Mushta and colleagues [2] carried out a comprehensive analysis of epidemiological research on CP in the Arab world, encompassing 32 studies carried out across seven Arab nations. Their findings suggest that the predominant type of CP is spastic quadriplegia. Only one study from Iraq was included in this systematic review that discussed services for children with CP. This study emphasizes how little is known about preventive care in Arab nations.

In Saudi Arabia, there are about 32 million individuals, with an average age of 22 years. Of these, 24.5% are children and 61.2% are men [20]. However, Saudi Arabia currently needs to enhance the thorough depiction of children with CP, taking into account their limitations and functional impairments. Numerous research works on CP in Saudi Arabia have been restricted to particular areas or cities [18,21]; other studies have mainly concentrated on the number of cases [22]. It is still unclear what traits children with CP have and what assistance is available to them. Policymakers should use this information to help them design services that are specifically suited to the requirements of families and children with CP.

The purpose of this study was to compare the growth patterns of children in Saudi Arabia who have CP with US and UK counterparts, according to data published by Wright et al. (2017) [16]. Our goal was to find out if it would be possible to track the growth of children with CP in Saudi Arabia by using growth charts designed for children with CP in the UK and the US. Furthermore, variations in growth patterns between the Saudi group and the British and American groups were evaluated while accounting for variables including the severity of CP and demographic traits. Our goals in conducting this comparative analysis were to further our understanding of global variations in the growth trajectories of this group and to aid in the creation of evidence-based care plans for children with CP in Saudi Arabia.

## 2. Materials and Methods

### 2.1. Participants

In order to investigate the growth patterns of Saudi Arabian children with CP, this study used an observational cross-sectional design. A retrospective chart review of CP children from the Children with Disability Association (CDA), Riyadh center, Saudi Arabia was conducted over two years (January 2021 to December 2022). The Children with Disability Association is a non-profit organization for children living with disabilities in Saudi Arabia, aiming to provide high-quality rehabilitation services across eleven centers.

### 2.2. Data Collection

The data collected were height, weight, head circumference, body mass index (BMI), and GMFCS level. A calibrated digital scale (standing, sitting, or wheelchair scale) was used to measure weight depending on the patient case. A stadiometer was used to measure standing height to the closest 0.1 cm, and for children who were unable to stand, a flexible rule was laid on the couch under the child for measurement. A non-stretchable tape was used to measure head circumference to the closest 0.1 cm. Following established protocols, healthcare professionals with training took these measurements.

Healthcare practitioners who are familiar with the GMFCS categorization method divided the participants into various GMFCS levels according to their gross motor function. The GMFCS has five levels. Level I: able to walk without restrictions, but limited in gross motor skills. Level II: the individual walks without assistance; there are limitations to their ability to walk outdoors and in the community. Level III: the individual walks with the assistance of mobility devices with limitations to walking in the community and outdoors. Level IV: limited self-mobility; children are transported or use power mobility in the community and outdoors. Level V: severe limitations in self-mobility despite the use of assistive technology, but not tube feeding [23].

### 2.3. Statistical Analysis

For each GMFCS level, descriptive statistics, such as means and standard deviations, were computed for head circumference, weight, height, and BMI. Analysis of variance (ANOVA) and post hoc comparisons were among the relevant statistical methods used in the comparative analyses to identify variations in growth patterns across various GMFCS levels.

### 2.4. Ethical Considerations

Informed consent from the participants’ parents or legal guardians was acquired prior to data collection. The Declaration of Helsinki’s ethical guidelines were followed when conducting this investigation. This study was approved by the Standing Committee of Bioethics Research (SCBR), Prince Sattam bin Abdulaziz University, Saudi Arabia (SCBR-051-2023). Throughout this study, participant data confidentiality and anonymity were guaranteed.

## 3. Results

A total of 107 children with a mean age of 5.04 years and SD of 3.25 were recruited for this study, including 73 males (68.2%), with the majority (86.0%) of the participants Saudi nationals. Regarding the CP type, the most prevalent subtype observed was spastic bilateral (41.1%), followed by spastic all limb—UL and LL (35.5%) and spastic unilateral—left (3.7%). Less common types included ataxic, dyskinetic, and hypotonic, highlighting the diverse clinical presentations within the study population. Furthermore, an analysis of GMFCS levels revealed that a substantial proportion of the participants were classified as Level III (25.2%) and Level IV (25.2%), indicating moderate to severe motor impairments with varying functional limitations.

Table 1 presents the descriptive statistics for weight, height, and BMI at assessment across different GMFCS levels among children with CP in the study cohort. The table illustrates the mean values and standard deviations for each parameter at each GMFCS level. There is notable variability in weight, height, and BMI across the different GMFCS levels. At GMFCS level I, children exhibit a higher mean weight (18.46 kg) and height (1.003 m) compared to the other levels, with a relatively low standard deviation, indicating less variability in these measurements within this group. Conversely, at GMFCS level V, children had the lowest mean weight (12.40 kg) and height (0.912 m), suggesting more significant impairments in growth and development among individuals with severe motor disabilities. The standard deviations across all GMFCS levels reflected the degree of variability in weight, height, and BMI within each group.

A one-way ANOVA was conducted to examine the effects of the GMFCS level on the weight, height, and BMI of children with CP. Significant differences were found in weight and BMI; F (4, 102) = 3.87, *p* < 0.01 and F (4, 102) = 2.86, *p* < 0.05, respectively. However, there was no statistically significant difference between height and the GMFCS level; F (4, 102) = 1.46, *p* = 0.22. A post hoc comparison using the Tukey test revealed that weight was significantly different between GMFCS level III and GMFCS level V (*p* = 0.01), and BMI was significantly different between GMFCS level III and GMFCS level IV (*p* = 0.04). 

Table 2 displays descriptive data on height, weight, and BMI at assessment for children with CP in all age groups for every GMFCS level divided by gender. The table is divided by two forms of sectioning: GMFCS Level I–III and GMFCS Level IV–V for the total sample, and male and female. Within each GMFCS level, the age categories are separated into 1–2 years, 2–4 years, 4–6 years, 6–8 years, 8–10 years, and 12–17 years. For each age group and GMFCS level combination, the table displays the weight, height, and BMI at assessment along with the mean, standard deviation, and number of observations. The results indicate that there are variations in anthropometric measurements among age groups within each GMFCS level. It draws attention to how children with CP have different growth trajectories according to the classification of their gross motor function.

Table 3 presents the z-scores for weight, height, and BMI at assessment across different GMFCS levels among children with CP. The z-scores represent the deviation of each individual’s measurement from the mean of a reference population in terms of standard deviations. Positive z-scores indicate measurements above the mean of the reference population, while negative z-scores indicate measurements below the mean. For weight at assessment, children classified under GMFCS Level I had a mean z-score of 0.48, indicating that their weights were, on average, 0.48 standard deviations above the mean of the reference population. Conversely, children in GMFCS Level V had a mean z-score of −0.36, suggesting that their weights were, on average, 0.36 standard deviations below the mean. Similar patterns were observed for height and BMI, with z-scores reflecting deviations from the reference population and mean across different GMFCS levels.

**Figure 1:**
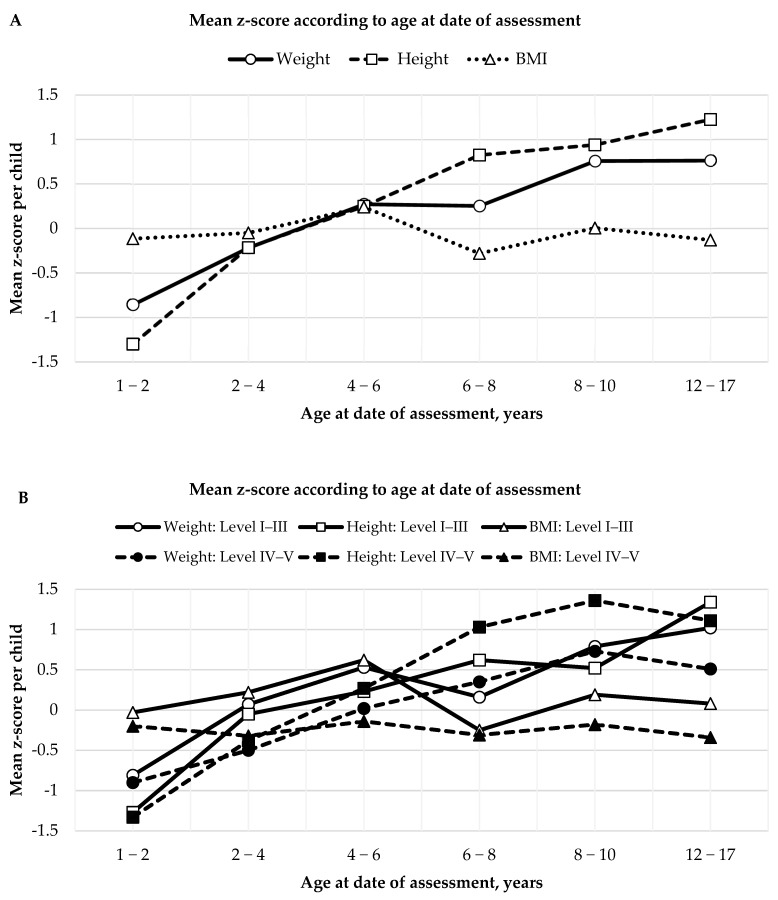
(**A**) mean z-scores of height, weight, and BMI of children with cerebral palsy in Saudi Arabia across age groups, and (**B**) mean z-scores presented based on GMFCS level across age groups (solid lines: level I–III; dashed lines: level IV–V).

Table 4 presents the z-scores for weight, height, and BMI at assessment across different age groups and GMFCS levels in children with CP. The table is divided into two sections based on GMFCS levels I–III and IV–V. Within each GMFCS level, the z-scores are further delineated across different age groups ranging from 1–2 years to 12–17 years. For GMFCS levels I–III, the mean z-scores for weight, height, and BMI generally tend to be positive, indicating values above the reference population mean. In contrast, for GMFCS levels IV–V, the mean z-scores are predominantly negative, suggesting values below the reference population mean. The standard deviations provide insights into the data variability within each subgroup, with higher values indicating a better dispersion from the mean. These z-scores enable the comparison of growth parameters across different GMFCS levels and age groups, facilitating a comprehensive understanding of growth patterns in children with CP. The results are illustrated in Figure 1.

Table 5 presents a comparison of height, weight, and BMI z-scores at various GMFCS levels in the UK, US, and Saudi Arabia. The z-scores show how far apart, in standard deviations, a given measurement is from the mean of the reference population.

## 4. Discussion

Crucial information about the growth trends of Saudi Arabian children with CP is provided by this cross-sectional study, which is to our knowledge the first study that has examined the growth patterns of children with CP in Saudi Arabia. Through the examination of anthropometric data gathered from 107 individuals at varying levels of the GMFCS, we have enhanced the comprehension of how children with CP develop physically in this area. Our findings demonstrate how children with various mobility disorders differ in terms of height, weight, and BMI, underscoring the significance of support and self-care for this population.

Physical growth and weight gain have been difficult for children with severe CP for many years [24]. Research has previously demonstrated a relationship between CP types and GMFCS levels with limited physical growth [25,26,27]. Physical growth limitations adversely affect the health and development of children with CP. Differences in growth patterns of children with CP were previously linked with feeding ability, motor function, and prematurity [28,29,30]. The severity of dysphagia has been found to be a significant predictor of poor growth for height, weight, and BMI [31]. Due to the lack of feeding data in our study, we were unable to analyze the impact of feeding disorders on the lower anthropometric data presented in this study.

Nonetheless, the results of our study are in agreement with those from previous research suggesting that height, weight, and BMI are greater in less severe cases (GMFCS level I to III) compared to more severe cases (GMFCS level IV and V) [32]. Additionally, younger children with CP exhibited lower heights, weights, and BMIs compared to their older counterparts as a result of the influence of their age. This is a similar trend to the growth charts of typically developing children. However, such growth charts, though they may be valuable for screening, are of limited significance when examining the growth patterns of children with CP and other chronic disabilities. Therefore, the need for improved growth pattern data in children with CP is of great importance to enhance clinical judgment and decision-making.

In prior studies [33,34], it has been shown that children with CP who have a growth deficiency have low levels of growth hormone (GH) and Insulin Growth Factor-1 (IGF-1) and are insufficiently responsive to insulin stimulation. In contrast, children with CP who have normal growth exhibit the same responses as peers who are typically developed in terms of their basal GH and IGF-1 levels. Despite this, they are unable to respond adequately to the insulin stimulation test. According to these findings, non-nutritional factors may contribute to the growth deficiency in children with CP. Accordingly, future research should focus more attention on the undefined role of growth hormones and growth patterns among children with CP.

Comparing the anthropometric data of the included sample with the growth charts of healthy Saudi children [35] revealed that the sample, in general, fell below the 50th percentile for height, weight, and BMI. However, the limited sample size and gender distribution in our study made it difficult to draw a valid comparison. Future research should investigate this issue and recruit a larger sample size to allow for accurate comparisons between children with CP and healthy children. The examination of z-scores for height, weight, and BMI in the US, Saudi Arabia, and the UK shed light on the growth patterns of children with CP across different populations and GMFCS levels. Overall, our findings imply that Saudi children with CP have greater height and weight z-scores than children in the US and the UK, suggesting possibly superior growth outcomes for this population. In different studies, it has been suggested that genes and the environment play a significant role in physical growth [36]. Genetic research in CP has made remarkable advances, significantly expanding our understanding of the disease. Various mutations and genetic alterations related to CP have been identified through whole-genome sequencing and exome sequencing [37,38].

It is important to note that despite this study’s novelty, it is not without limitations. First, there was a small sample size included in this study compared to previously published studies [16,32], so caution should be exercised when interpreting the results. Researchers have difficulty identifying potential CP candidates in the Kingdom of Saudi Arabia due to the lack of a CP registry. A larger sample size and population-based analysis should be considered for future research. Second, although comparing growth patterns between different countries is important, it may be misleading due to genetic, environmental, geographical, socioeconomic, and racial differences. Third, to our knowledge, this is the first study of growth patterns of children with CP in the Arab world. The absence of similar data from Arab countries makes it difficult to draw recommendations regarding healthcare systems at the regional level and inform policymakers. Finally, physical growth and development are significantly affected by the CP phenotype, prior surgeries, and family history. These factors were not included in our study and should be considered in future research.

## 5. Conclusions

The current research shows that the growth patterns of children with CP differ significantly from those of healthy children. The influences of age and the GMFCS level were presented and discussed. We conclude that the physical growth and development of children with CP will depend on their age and gross motor development. These findings can serve as guidelines for the development of targeted treatments and services intended to maximize the growth and well-being of children with CP, as well as inform Saudi Arabian educators, policymakers, and healthcare professionals. Finally, this study sheds light on the urgent need for national growth percentiles for children with CP and chronic disorders.

## Figures and Tables

**Table 1:** Descriptive statistics of weight, height, and BMI at assessment across different GMFCS levels among children with CP.

GMFCS Level	Weight at Assessment (kg)	Height at Assessment (m)	BMI
Level I	18.463 ± 9.30	1.003 ± 0.08	18.446 ± 9.24
Level II	13.500 ± 3.73	0.949 ± 0.114	14.815 ± 2.31
Level III	18.696 ± 10.16	1.005 ± 0.177	17.998 ± 8.30
Level IV	13.659 ± 4.24	0.970 ± 0.120	14.339 ± 2.72
Level V	12.400 ± 5.67	0.912 ± 0.182	14.375 ± 2.07

Data presented as mean ± standard deviation, GMFCS = Gross Motor Function Classification System, BMI = body mass index, kg = kilogram, m = meter.

**Table 2:** Descriptive data on children with CP’s weight, height, and BMI at evaluation for each GMFCS level across several age groups and by gender.

GMFCS Level	Age (y)	N	Weight at Assessment (kg)	Height at Assessment (m)	BMI
Total sample					
I–III	1–2	7	9.33 ± 0.78	0.77 ± 0.05	15.80 ± 2.89
	2–4	13	14.90 ± 8.19	0.93 ± 0.09	16.91 ± 7.53
	4–6	15	16.91 ± 3.22	1.01 ± 0.04	16.41 ± 2.74
	6–8	9	19.72 ± 11.80	1.02 ± 0.06	19.51 ± 14.2
	8–10	8	22.65 ± 12.01	1.14 ± 0.22	16.21 ± 3.87
	12–17	3	17.33 ± 4.25	1.04 ± 0.15	16.01 ± 2.19
	Total	55	16.82 ± 8.49	0.98 ± 0.14	16.90 ± 6.98
IV–V	1–2	5	8.36 ± 1.71	0.75 ± 0.07	14.81 ± 3.98
	2–4	22	10.36 ± 2.93	0.85 ± 0.09	14.10 ± 2.99
	4–6	12	14.93 ± 2.64	0.99 ± 0.07	15.01 ± 1.06
	6–8	5	16.21 ± 3.16	1.08 ± 0.09	13.52 ± 0.65
	8–10	6	18.34 ± 3.44	1.13 ± 0.05	14.21 ± 1.67
	12–17	2	21.35 ± 15.20	1.16 ± 0.35	14.30 ± 2.51
	Total	52	13.12 ± 4.97	0.94 ± 0.15	14.40 ± 2.41
Male					
I–III	1–2	5	9.48 ± 0.84	0.75 ± 0.05	16.80 ± 2.84
	2–4	8	17.42 ± 9.69	0.97 ± 0.07	18.31 ± 9.48
	4–6	11	18.16 ± 2.95	1.03 ± 0.04	17.12 ± 2.79
	6–8	6	20.50 ± 14.72	0.99 ± 0.06	21.50 ± 17.50
	8–10	8	22.63 ± 12.02	1.14 ± 0.22	16.22 ± 3.87
	12–17	1	21.53 ± 0.00	1.12 ± 0.00	17.11 ± 0.00
	Total	39	18.22 ± 9.48	1.01 ± 0.15	17.80 ± 8.08
IV–V	1–2	2	7.30 ± 1.84	0.78 ± 0.13	11.90 ± 1.19
	2–4	13	9.84 ± 3.36	0.83 ± 0.09	14.12 ± 3.70
	4–6	9	15.12 ± 3.03	1.01 ± 0.07	14.80 ± 0.93
	6–8	3	16.30 ± 0.57	1.11 ± 0.02	13.32 ± 0.22
	8–10	5	18.72 ± 3.64	1.14 ± 0.05	14.31 ± 1.86
	12–17	2	21.29 ± 15.20	1.16 ± 0.35	14.30 ± 2.51
	Total	34	13.64 ± 5.65	0.96 ± 0.16	14.11 ± 2.51
Female					
I–III	1–2	2	8.95 ± 0.63	0.82 ± 0.05	13.31 ± 0.02
	2–4	5	10.89 ± 2.21	0.86 ± 0.08	14.70 ± 1.67
	4–6	4	13.80 ± 1.28	0.97 ± 0.04	14.41 ± 1.48
	6–8	3	18.01 ± 3.75	1.07 ± 0.04	15.63 ± 2.36
	8–10	0	0.00 ± 0.00	0.00 ± 0.00	0.00 ± 0.00
	12–17	2	15.30 ± 3.18	1.00 ± 0.17	15.43 ± 2.76
	Total	16	13.31 ± 3.68	0.94 ± 0.11	14.71 ± 1.72
IV–V	1–2	3	9.07 ± 1.50	0.74 ± 0.05	16.70 ± 4.18
	2–4	9	10.90 ± 2.20	0.87 ± 0.07	14.21 ± 1.71
	4–6	3	14.21 ± 0.76	0.94 ± 0.06	15.90 ± 1.05
	6–8	2	15.61 ± 6.22	1.05 ± 0.04	13.81 ± 1.17
	8–10	1	16.00 ± 0.00	1.07 ± 0.00	14.01 ± 0.00
	12–17	0	0.00 ± 0.00	0.00 ± 0.00	0.00 ± 0.00
	Total	18	12.01 ± 3.21	0.89 ± 0.12	14.80 ± 2.21

Data presented as mean ± standard deviation, GMFCS = Gross Motor Function Classification System, BMI = body mass index, y = year, N = number, kg = kilogram, m = meter.

**Table 3:** Z-scores for weight, height, and BMI at assessment across GMFCS levels in children with cerebral palsy.

GMFCS Level	N	Z-Score: Weight at Assessment (kg)	Z-Score: Height at Assessment (m)	Z-Score: BMI
Level I	8	0.48 ± 1.29	0.26 ± 0.53	0.51 ± 1.71
Level II	20	−0.20 ± 0.52	−0.10 ± 0.76	−0.16 ± 0.43
Level III	27	0.52 ± 1.41	0.27 ± 1.18	0.43 ± 1.53
Level IV	27	−0.18 ± 0.59	0.04 ± 0.80	−0.25 ± 0.50
Level V	25	−0.36 ± 0.79	−0.34 ± 1.21	−0.24 ± 0.38

Data presented as mean ± standard deviation, GMFCS = Gross Motor Function Classification System, BMI = body mass index, y = year, N = number, kg = kilogram, m = meter.

**Table 4:** Z-scores for height, weight, and BMI during evaluation in relation to GMFCS levels and age groups in children with cerebral palsy.

GMFCS Level	Age (y)	N	Z-Score: Weight at Assessment (kg)	Z-Score: Height at Assessment (m)	Z-Score: (BMI)
I–III	1–2	7	−0.81 ± 0.13	−1.27 ± 0.36	−0.03 ± 0.52
	2–4	13	0.07 ± 1.09	−0.05 ± 0.57	0.22 ± 1.40
	4–6	15	0.53 ± 1.20	0.23 ± 0.30	0.62 ± 1.86
	6–8	9	0.16 ± 0.44	0.62 ± 0.29	−0.25 ± 0.36
	8–10	8	0.79 ± 2.23	0.52 ± 1.79	0.19 ± 1.03
	12–17	3	1.02 ± 1.05	1.34 ± 1.18	0.08 ± 0.32
	Total	55	0.25 ± 1.18	0.14 ± 0.97	0.23 ± 1.29
IV–V	1–2	5	−0.90 ± 0.20	−1.33 ± 0.39	−0.20 ± 0.46
	2–4	22	−0.50 ± 0.42	−0.38 ± 0.47	−0.32 ± 0.64
	4–6	12	0.02 ± 0.34	0.27 ± 0.46	−0.14 ± 0.22
	6–8	5	0.35 ± 0.24	1.03 ± 0.29	−0.31 ± 0.15
	8–10	6	0.73 ± 0.59	1.36 ± 0.33	−0.18 ± 0.42
	12–17	2	0.51 ± 1.28	1.11 ± 1.37	−0.34 ± 0.31
	Total	52	−0.26 ± 0.69	−0.14 ± 1.02	−0.24 ± 0.45

Data presented as mean ± standard deviation, GMFCS = Gross Motor Function Classification System, BMI = body mass index, y = year, N = number, kg = kilogram, m = meter.

**Table 5:** Comparison of z-scores for height, weight, and BMI among children with cerebral palsy at different GMFCS levels in the UK, US, and Saudi Arabia.

	UK			US			Saudia Arabia		
	Height	Weight	BMI	Height	Weight	BMI	Height	Weight	BMI
GMFCS Level	Mean ± SD	Mean ± SD	Mean ± SD	Mean ± SD	Mean ± SD	Mean ± SD	Mean ± SD	Mean ± SD	Mean ± SD
I	−0.63 ± 1.07	−0.3 ± 1.23	0.09 ± 1.28	0.38 ± 0.54	0.07 ± 0.79	−0.34 ± 0.67	0.26 ± 0.53	0.48 ± 1.29	0.51 ± 1.71
II	−1.03 ± 1.28	−0.37 ± 1.50	0.37 ± 1.38	0.37 ± 0.61	0.25 ± 0.94	−0.13 ± 0.73	−0.10 ± 0.76	−0.20 ± 0.52	−0.16 ± 0.43
III	−0.86 ± 1.14	−0.59 ± 1.53	0.07 ± 1.59	0.80 ± 0.56	0.50 ± 0.86	−0.12 ± 0.79	0.27 ± 1.18	0.52 ± 1.41	0.43 ± 1.53
IV	−1.79 ± 1.43	−1.63 ± 1.95	−0.41 ± 1.96	0.49 ± 0.62	0.20 ± 1.02	−0.18 ± 0.82	0.04 ± 0.80	−0.18 ± 0.59	−0.25 ± 0.50
V	−1.68 ± 1.56	−1.52 ± 1.77	−0.80 ± 1.75	0.71 ± 0.62	0.62 ± 0.85	−0.26 ± 0.74	−0.34 ± 1.21	−0.36 ± 0.79	−0.24 ± 0.38

## Data Availability

The original contributions presented in this study are included in the article, and further inquiries can be directed to the corresponding author.
